# 

**Published:** 1879-10

**Authors:** 


SURG GENLS OFFS

Vol. XV. OCTOBER, 1879. No. 3.

### THE BISTOURY:

### A QUARTERLY JOURNAL,

*Devoted to the Exposition of Charlatanism in Medicine and the Education of the People upon Medical Subjects!*

NOTHING TO INDEX

Thad S. Up de Graff, M. D., Editor.

*Terms of Subscription, Fifty Cents per Annum, in Advance*

ELMIRA, N. Y.

PRINTED FOR THE PROPRIETOR, AT THE DAILY ADVERTISER JOB PRINTING HOUSE.

